# Transcriptomic Profiles Reveal the Interactions of Cd/Zn in Dwarf Polish Wheat (*Triticum polonicum* L.) Roots

**DOI:** 10.3389/fphys.2017.00168

**Published:** 2017-03-23

**Authors:** Yi Wang, Xiaolu Wang, Chao Wang, Fan Peng, Ruijiao Wang, Xue Xiao, Jian Zeng, Houyang Kang, Xing Fan, Lina Sha, Haiqin Zhang, Yonghong Zhou

**Affiliations:** ^1^Triticeae Research Institute, Sichuan Agricultural UniversityWenjiang, China; ^2^Key Laboratory of Crop Genetic Resources and Improvement, Ministry of Education, Sichuan Agricultural UniversityWenjiang, China; ^3^College of Resources, Sichuan Agricultural UniversityWenjiang, China

**Keywords:** dwarf polish wheat, RNA-Seq, cadmium, zinc, interaction

## Abstract

Different intra- or interspecific wheat show different interactions of Cd/Zn. Normally, Zn has been/being widely utilized to reduce the Cd toxicity. In the present study, the DPW seedlings exhibited strong Cd tolerance. Zn and Cd mutually inhibited their uptake in the roots, showed antagonistic Cd/Zn interactions. However, Zn promoted the Cd transport from the roots to shoots, showed synergistic. In order to discover the interactive molecular responses, a transcriptome, including 123,300 unigenes, was constructed using RNA-Sequencing (RNA-Seq). Compared with CK, the expression of 1,269, 820, and 1,254 unigenes was significantly affected by Cd, Zn, and Cd+Zn, respectively. Only 381 unigenes were co-induced by these three treatments. Several metal transporters, such as *cadmium-transporting ATPase* and *plant cadmium resistance 4*, were specifically regulated by Cd+Zn. Other metal-related unigenes, such as *ABC transporters, metal chelator, nicotianamine synthase* (*NAS*), *vacuolar iron transporters* (*VIT*), *metal-nicotianamine transporter YSL* (*YSL*), and *nitrate transporter* (*NRT*), were regulated by Cd, but were not regulated by Cd+Zn. These results indicated that these transporters participated in the mutual inhibition of the Cd/Zn uptake in the roots, and also participated in the Cd transport, accumulation and detoxification. Meanwhile, some unigenes involved in other processes, such as oxidation-reduction, auxin metabolism, glutathione (GSH) metabolism nitrate transport, played different and important roles in the detoxification of these heavy metals.

## Introduction

In plants, non-essential heavy metals cause toxicity and eventually inhibit plant growth and development (Balen et al., 2011). Cadmium (Cd), a heavy metal widespread in the environment, damages the photosynthetic apparatus, affects the respiratory and nitrogen metabolism, and alters the balance of water and nutrient uptake (Herbette et al., 2006; Balen et al., 2011). Cd absorbed by plants can be introduced into the food chain (McLaughlin et al., 1999). Consumption, either directly or indirectly, of these parts could be a human health concern (Grant et al., 2008). Therefore, the Cd concentrations in several safe cereal grains were limited below 0.2 mg/Kg. In contrast, zinc (Zn) is an essential metal for plant growth. It has been/being widely utilized to reduce the Cd toxicity, although the effect varies with genotypes, the dose and duration of the Zn and Cd exposure (Rizwan et al., 2016). Excess Zn also limits the plant growth and causes a strong toxicity (Zhao et al., 2005; Wang et al., 2009). Plants therefore need to prevent damage from non-essential metals and ensure the proper homeostasis of essential metals (Lin and Aarts, 2012).

Cd and Zn, co-existed in the soil, cause various synergistic and antagonistic interactions according to the species, external metal concentrations, tissues, and developmental stages (Cataldo et al., 1983; Nan et al., 2002; Hassan et al., 2005; Sun et al., 2005; Balen et al., 2011; Cherif et al., 2011; Tkalec et al., 2014). In soybean, the uptake of Cd/Zn exhibits competitive inhibition (Cataldo et al., 1983). In durum and bread wheat, Cd and Zn mutually inhibit their uptake in the roots, stems and leaves (Hart et al., 2002, 2005). In tomato (Cherif et al., 2011) and *Lemna minor* (Balen et al., 2011), Zn inhibits the Cd uptake. However, the Cd/Zn interactions are not always antagonistic. Synergistic interactions were observed in both wheat and corn under field conditions (Nan et al., 2002). In tobacco, Zn promotes the Cd uptake, while Cd inhibits the Zn uptake in the roots and leaves (Tkalec et al., 2014). In rice, Zn increases the Cd concentration in the shoots, but inhibits the Cd uptake in the roots (Hassan et al., 2005). However, all these studies focused on the transport and biochemical responses by measuring plant growth, metal concentration, pigment content, and antioxidant content (Cataldo et al., 1983; Nan et al., 2002; Hassan et al., 2005; Sun et al., 2005; Cherif et al., 2011; Balen et al., 2011; Tkalec et al., 2014). Meanwhile, studies revealed the changes of transcriptomic profiles were focused on Cd or Zn alone (Herbette et al., 2006; Di Baccio et al., 2011; Lin et al., 2013). The changes of transcriptomic profiles for Cd/Zn interactions were not revealed.

Transcriptomes of *Triticum turgidum* (2n = 4x = 28, AABB) and common wheat (2n = 6x = 42, AABBDD) using RNA-sequencing (RNA-Seq) have been reported (Duan et al., 2012; Schreiber et al., 2012; Krasileva et al., 2013). Recently, the transcriptomic profiles of the developing starchy endosperm and the grain filling of bread wheat, and the dwarfism of dwarf Polish wheat were revealed using RNA-Seq (Pont et al., 2011; Pellny et al., 2012; Wang et al., 2016a). Although the molecular responses to Cd or Zn in plants have been widely investigated (Herbette et al., 2006; Di Baccio et al., 2011; Lin et al., 2013), the similar study in wheat using RNA-Seq is not processed. Based on genetic analysis and taxonomical classification, Polish wheat (2n = 4x = 28, AABB, *Triticum polonicum* L.) presents a low level of genetic similarity with *T. durum, T. turgidum*, and *T. aestivum* (Wang et al., 2013; Michalcová et al., 2014). Due to the high thousand kernel weights and high Zn, Fe, and Cu concentrations in the seeds (Wiwart et al., 2013), and the dwarfing gene (Kang et al., 2012), Polish wheat has attracted the interest of producers and breeders (Wiwart et al., 2013). Dwarf polish wheat (DPW, *Triticum polonicum* L.) which collected from Tulufan, Xingjiang, China, shows high tolerance to Cd and Zn. Therefore, it is a desirable material for studying Cd/Zn interactions. Previously proteomic study revealed that many proteins mainly participated in sucrose, glutathione (GSH), S-adenosyl-l-methionine (SAM), organic acids metabolisms and oxidation-reduction process were response to the Cd/Zn interactions on two days after treatments (Wang et al., 2016b). However, results of transtriptomic and proteomic analysis are very low overlay. It is interesting to investigate that what kinds of genes response to the Cd/Zn interactions when prolonged the treated time. In the present study, our aims are therefore to investigate the transcriptome responses under Cd, Zn and Cd+Zn stresses, finally reveal the molecular mechanisms of Cd/Zn interactions in the DPW roots on 5 days after treatments.

## Materials and methods

### Plant material and growth conditions

Seeds of DPW were sterilized with 1% NaOCl. After germination at room temperature for 5 days, the seedlings with plastic foam support grown on distilled water for 3 days and then were cultured in nutrient solution (Hoagland's Modified Basal Salt Mixture, MP Biomedicals, USA) in a growth chamber at 25°C with a relative humidity 70% under a 16-h-light/8-h-dark cycle. Per 50 plants were cultured in a container which contained 8 l nutrient solution with pH 6.0. The nutrient solution was refreshed every 5 days. Two-leaf seedlings were stressed with control (CK, null), 40 μM CdSO_4_ (Cd), 800 μM ZnCl_2_ (Zn, the Zn concentration of arable soil varies from 25 to 150 mg/Kg), and 40 μM CdSO_4_+ 800 μM ZnCl_2_ (Cd+Zn). On 5 days after treatments, the roots collected from 15 plants (15 plants per biological replicate, three biological replicates) were snap frozen in liquid nitrogen and stored at −80°C for RNA-Seq.

### Phenotype characterization

On 5 days after treatments, the leaves and roots were collected from 20 plants (20 plants per biological replicate, three biological replicates). The roots were successively washed with 0.1 μM EDTA and ddH_2_O. The length of the longest root and leaf per plant were measured. Their fresh and dry weights were also determined. The percentage of leaf or root dry weight was calculated as (leaf or root dry weight of 20 plants)/(total dry weight of 20 plants) ×100%; the percentage of leaf or root fresh weight was calculated as (leaf or root fresh weight of 20 plants)/(total fresh weight of 20 plants) × 100%. After weighing, all tissues were dried at 80°C for 2 days to measure metal concentration. At the same time, the percentages of water content and dry weight were calculated. All data analysis (student's *t*-test) was performed with SPSS 20.0 and figures were drawn with Sigmaplot 12.0.

### Analysis of Cd and Zn contents

The Cd and Zn concentrations were measured as described by Wang et al. (2014). Reference standard solutions of Cd and Zn were purchased from the Fisher Scientific Ltd. (Shanghai, China). All data analysis (student's *t*-test) was performed with SPSS 20.0 and figures were drawn with Sigmaplot 12.0.

### RNA isolation

Total RNA of each sample (null, Cd, Zn and Cd+Zn) was isolated using the E.Z.N.A.® Total RNA Kit II (Omega, USA). The RNA was checked for quality on 1% agarose gels and the NanoPhotometer® spectrophotometer (Implen, Germany) and the RNA 6000 Nano Assay Kit of the Bioanalyzer 2100 system (Agilent Technologies, USA). The Qubit® RNA Assay Kit in Qubit® 2.0 Flurometer (Life Technologies, Shanghai, China) was used to measure RNA concentration.

### Library construction and sequencing

mRNA was purified from total RNA using poly-T oligo-attached magnetic beads (Life Technologies, USA) and transcribed to cDNA using random oligonucleotides and M-MuLV Reverse Transcriptase (RNase H^−^) (TaKaRa, Dalian, China). NEBNext adaptor oligonucleotides (Illumia, USA) were ligated to 3′ ends of cDNA fragments. Then, 200-bp cDNA fragments were purified using the AMPure XP beads system (Beckman Coulter, USA). Ten cycles of PCR amplifications were performed to enrich cDNA fragments using the NEB Universal PCR primer and Index primer (Illumia, USA). The PCR products were purified using the AMPure XP beads system and quantified using the Agilent Bioanalyzer 2100 system. Finally, the four-coded samples were clustered by a cBot Cluster Generation System using the TruSeq PE Cluster Kit v3-cBot-HS (Illumia, USA), and then sequenced on an Illumina Hiseq 2000 platform.

### Transcriptome assembly

Adapter reads containing poly-N and low-quality reads were removed using Novogene-written perl scripts to product clean reads. The paired-end clear reads generated contigs using Trinity (V2012-10-15) (Grabherr et al., 2011) with minimum K-mer coverage was 2, and other parameters were default.

### Unigenes functional annotation

The putative unigene function was annotated using a series of databases, including BLASTx against the NCBI NR and NT, Swiss-Prot databases, the Kyoto Encyclopedia of Genes and Genomes (KEGG), Ortholog database (KO) and Clusters of Orthologous Groups of proteins (KOG/COG) database, with an *E*-value cutoff of 10-6, hidden Markov models scan (hmmscan) against the protein family (Pfam) (Eddy, 2011), and Blast2GO against Gene Ontology (GO) (Götz et al., 2008). Functional categories of putative unigenes were grouped using the GO database, KEGG database, and KOG database.

### Differential expression analysis

Clean reads were aligned against reference transcript sequences to produce a read count using the RSEM package (Li and Dewey, 2011). The read counts of each unigene were converted into RPKM values to normalize the gene expression (Mortazavi et al., 2008). Differentially expressed genes (DEGs) were calculated using the DEseq method (Ander and Huber, 2010).

### Quantitative real-time PCR (qPCR) for validation of partial DEGs

qPCR and data analysis were performed as described by Wang et al. (2015). Twelve differential expressed genes were validated and their primers were listed in STable 1. *Actin* (Wang et al., 2015) was used to standardize transcript levels in each sample. The ΔΔCt method was used to normalize the relative expression of each gene using the software of Bio-Rad CFX manager v. 1.6.541.1028. The student's *t*-test (*P* < 0.05) was conducted for the evaluation of significance of mean values.

## Results

### Wheat growth

**Figure 1:**
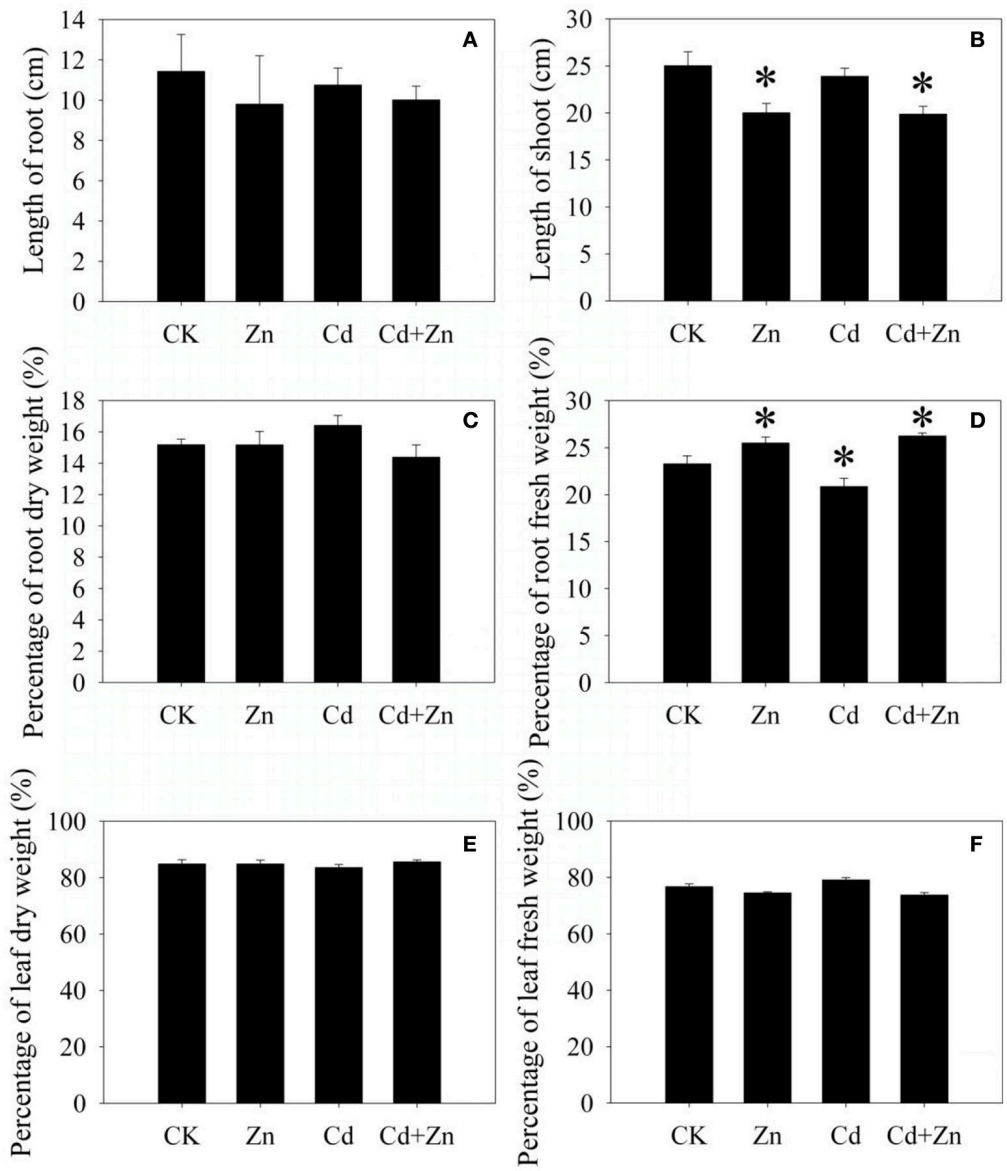
**Growth of DPW exposed to Cd, Zn and Cd+Zn**. **(A)** root length; **(B)** shoot length; **(C)** weight percentages of dry roots; **(D)** weight percentages of fresh roots; **(E)** weight percentages of dry leaves; **(F)** weight percentages of fresh leaves. Values were means ± standard error (three biological replicates); asterisk represented significant difference (*P* < 0.05).

Compared with control (CK), Cd did not inhibit the root and shoot growth after 5 days of treatment (Figures 1A,B). Excess Zn and Zn + Cd slightly reduced the root length (Figure 1A) and significantly (*P* < 0.05) inhibited the shoot growth (Figure 1B). Compared with CK, Cd significantly reduced the fresh root weight percentage (Figure 1D), but did not affect the dry root weight percentage (Figure 1C) and the dry and fresh leaf weight percentage (Figures 1E,F). Zn and Cd+Zn significantly increased the fresh root weight percentage (Figure 1D), but did not affect the leaf weight percentage (Figures 1E,F). The results described above indicated that metal stresses obviously affected the plant growth.

### Cd and Zn mutually inhibited their uptake in the roots

**Figure 2:**
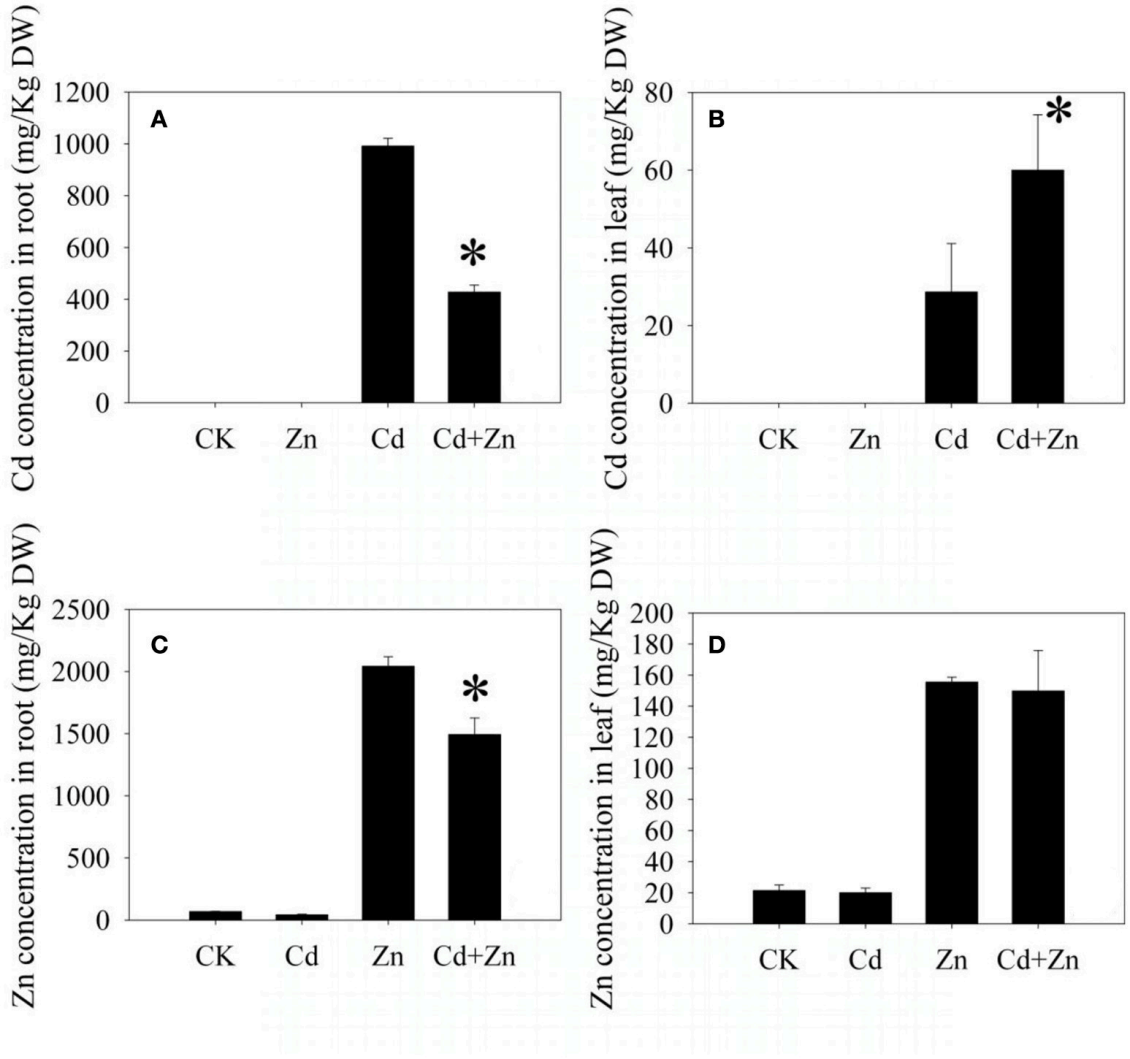
**Metal concentrations in the roots and leaves**. **(A,B)** Cd concentrations; **(C,D)** Zn concentrations. Values were means ± standard error (three biological replicates); asterisk represented significant difference (*P* < 0.05).

After 5 days of treatments, an accumulation of Cd was not observed in all investigated samples which were unexposed to Cd (CK and Zn, Figures 2A,B). The Cd concentration in the roots under Cd+Zn stress was significantly lower (*P* < 0.01) than that under Cd stress (Figure 2A). However, opposite result was observed in the shoots (Figure 2B). These results indicated that Zn inhibited the Cd uptake in the roots, but promoted the Cd transport from the roots to shoots.

The Zn concentration was higher in the roots than that in the leaves (Figures 2C,D). In the roots, the Zn concentration under Cd+Zn stress was significantly lower (*P* < 0.01) than that under Zn stress (Figure 2C). In the leaves, the Zn concentrations were similar between Zn stress and Cd+Zn stress (Figure 2D). Thus, Cd only inhibited the Zn uptake in the roots.

### *De novo* assembly and functional annotation

RNA-Seq generated 18.35 Gb nucleotides. All raw read sequences were deposited to the NCBI Sequence Read Archive (SRA) database with accession numbers SRR2973581, SRR2973582, SRR2973583, and SRR2973584. Approximately 123,300 unigenes that varied from 201 bp to 16,390 bp (mean length was 660 bp, N50 value was 870 bp) were assembled. Amino acid (AA) sequences of 76,395 (61.96%) unigenes were predicted.

Though Blastx against several public databases, 84,709 (68.70%) unigenes were annotated. Among these annotated unigenes, 63,221 unigenes were functionally classified in GO; 13,637 unigenes were classified into 26 KOG categories; 10,576 unigenes were functionally classified in KEGG. All data of sequences and functional annotation were deposited to the NCBI Transcriptome Shotgun Assembly (TSA) database with accession number GEDP00000000.

### Cd-, Zn-, and Cd+Zn- induced DEGs

Cd and Zn mutually inhibited their uptake in the roots, Zn promoted the Cd transport from the roots to shoots (Figure 2), and these treatments also affected growth (Figure 1). Theoretically, some unigenes participated in the interactions should be regulated by these treatments, which were revealed using RNA-Seq.

**Figure 3:**
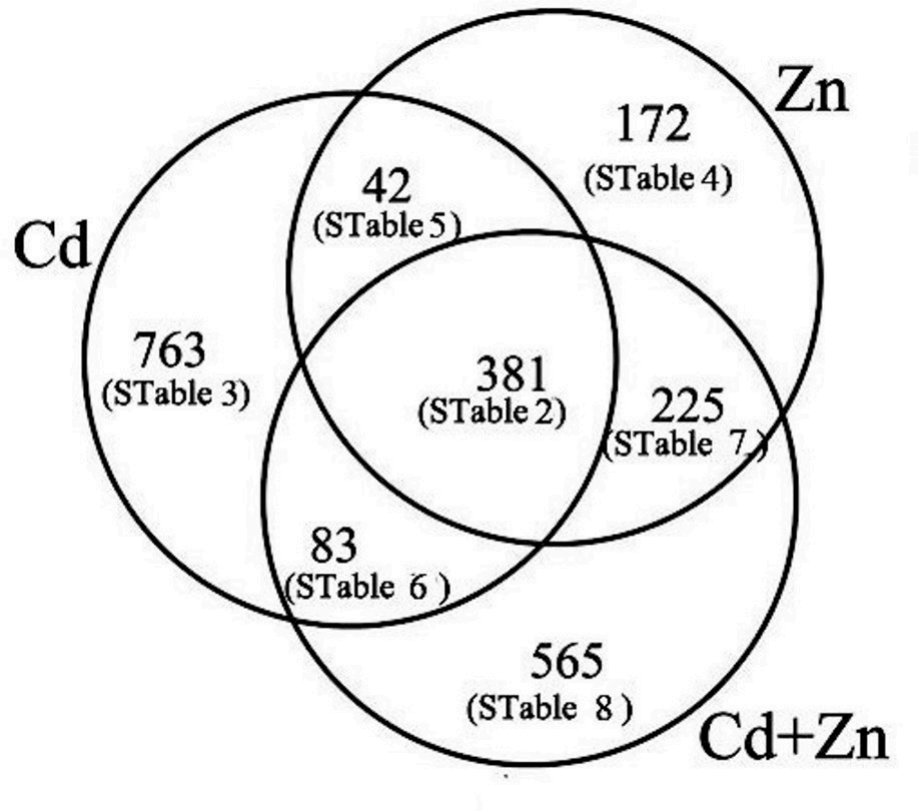
**Numbers of DEGs were classfied into differential interactions of Cd/Zn**.

Compared with CK, the expression of 1,269, 1,254, and 820 unigenes was changed by Cd, Cd+Zn, and Zn, respectively (Figure 3). Among these DEGs, the expression of 381 unigenes mainly participated in several basic processes were co-changed by Cd, Zn, and Cd+Zn (STable 2), suggesting that there were differential molecular responses to Cd, Zn, and Cd+Zn stresses.

The remaining DEGs were arranged into 6 subgroups (Figure 3).

1. The expression of 763 DEGs, which consisted of 96 down- and 667 up-regulated unigenes, was changed by Cd, but was not affected by Zn and Cd+Zn (log_2_fold-changes of CK/Cd were >1 or <−1 with P values below 1.00E-05, log_2_fold-changes of CK/Zn and CK/Cd+Zn varied from 1 to −1, Figure 3, STable 3). These DEGs mainly participated in glutathione (GSH) metabolism (4 down- and 7 up-), oxidation-reduction process (1 down- and 12 up-), carbohydrate metabolism (3 down- and 19 up-), metal transport (9 up-), nitrate metabolism (3 up- and 1 down-), and metal chelation (2 up-) (Table 1).
2. 172 Zn-induced DEGs, which consisted of 36 down- and 136 up-regulated unigenes, were not affected by Cd and Cd+Zn (log_2_fold_changes of CK/Zn were >1 or <−1 with *P*-values below 1.00E-05, log_2_fold-changes of CK/Cd, and CK/Cd+Zn varied from 1 to −1, Figure 3, STable 4). Among them, several noteworthy DEGs were correspondingly grouped into carbohydrate metabolism (3 down- and 8 up-), GSH metabolism (4 up-), and oxidation-reduction process (3 up-) (Table 1).
3. The expression of 42 DEGs was changed by Cd and Zn, but was not changed by Cd+Zn (log_2_-fold changes of CK/Cd and CK/Zn were >1 or <−1 with *P*-values below 1.00E-05, log_2_fold-changes of CK/Cd+Zn varied from 1 to −1, Figure 3, STable 5), which suggested that the expression of these DEGs was mutually suppressed by Cd and Zn under Cd+Zn stress. Among these unigenes, several noteworthy DEGs participated in GSH and carbohydrate metabolism, respectively (Table 1).
4. The expression of 83 DEGs which included 51 down- and 32 up-regulated unigenes was changed by Cd and Cd+Zn, but was not changed by Zn (log_2−fold_ changes of CK/Cd and CK/Cd+Zn were >1 or <−1 with *P*-values below 1.00E-05, log_2_fold-changes of CK/Zn varied from 1 to −1, Figure 3, STable 6), suggesting that these DEGs were specifically Cd-induced. Among these unigenes, some noteworthy unigenes participated in carbohydrate metabolism (2 up-), oxidation-reduction process (*peroxidase 15* and *catalase isozyme 2*), and nitrate transport (*nitrate transporter 1.5*) (Table 1).
5. The expression of 225 DEGs, consisting of 174 down- and 51 up-regulated unigenes, was affected by Zn and Cd+Zn, but was not affected by Cd alone (log_2_-fold changes of CK/Zn and CK/Cd+Zn were >1 or <−1 with *P*-values below 1.00E-05, log_2_fold-changes of CK/Cd varied from 1 to −1, Figure 3, STable 7), suggesting that these DEGs were specifically Zn-induced. Among them, noteworthy DEGs mainly participated in oxidation-reduction process (3 up-), carbohydrate metabolism (2 down-), GSH metabolism (1 up-) and nitrate metabolism (1 up-) (Table 1).
6. Additionally, the expression of 565 DEGs which consisted of 95 down- and 470 up-regulated unigenes was affected by Cd+Zn, but was not affected by Cd and Zn (log_2_-fold changes of CK/Cd+Zn were >1 or <−1 with *P*-values below 1.00E-05, log_2_fold-changes of CK/Zn and CK/Cd varied from 1 to −1, Figure 3, STable 8), suggesting that these DEGs specifically responded to Cd+Zn. Among these DEGs, some noteworthy DEGs participated in carbohydrate metabolism (2 down- and 9 up-), metal transport (2 up-) and nitrate transport (2 down-) (Table 1).

**Table 1:** **Noteworthy DEGs in different groups**.

**(1) Cd-induced DEGs were not induced by Zn and Cd+Zn**			
**Unigene number**	**Annotation**	**Fold change^a^**	**Metabolism type**
comp229495_c0	Peroxidase 1	2.09	Oxidation-reduction process
comp186643_c0	Peroxidase 12	1.70	
comp132618_c0	Peroxidase 2	4.29	
comp145649_c0	Peroxidase 2	3.59	
comp228081_c0	Peroxidase 2	2.35	
comp258530_c0	Peroxidase 2	2.95	
comp232606_c0	Peroxidase 2	1.24	
comp257733_c1	Peroxidase 39	2.43	
comp256271_c1	Peroxidase 4	1.76	
comp263129_c1	Peroxidase 47	2.35	
comp268022_c0	Peroxidase 47	3.21	
comp259330_c1	Peroxidase 5	−1.39	
comp265682_c0	Peroxidase N	3.43	
comp267415_c0	Disulfide isomerase-like 1-4	1.67	GSH metabolism
comp167112_c0	Glutaredoxin-C2	3.42	
comp270080_c0	Glutathione S-transferase 1	−1.02	
comp262123_c0	Glutathione S-transferase 2	1.66	
comp258739_c0	Glutathione S-transferase 3	−1.49	
comp249955_c0	Glutathione S-transferase GSTF1	−1.48	
comp256282_c1	Glutathione S-transferase GSTU1	−1.04	
comp169916_c0	Glutathione S-transferase	1.33	
comp249889_c0	Hydroxyacylglutathione hydrolase 3	1.01	
comp247586_c0	Lactoylglutathione lyase	1.65	
comp244017_c0	S-formylglutathione hydrolase	3.05	
comp257426_c0	Callose synthase 1	1.50	Carbohydrate metabolism
comp255049_c0	Callose synthase 2	2.33	
comp269308_c0	Callose synthase 3	1.17	
comp268926_c1	Callose synthase 8	1.57	
comp267574_c1	Cellulose synthase A catalytic subunit 1	1.27	
comp258633_c0	Cellulose synthase A catalytic subunit 1	1.79	
comp267508_c0	Cellulose synthase A catalytic subunit 5	1.10	
comp263049_c0	Cellulose synthase-like protein E2	1.46	
comp260194_c0	Soluble starch synthase 3	1.77	
comp267559_c0	Fructose-bisphosphate aldolase	−1.04	
comp256169_c0	Alpha-galactosidase	1.33	
comp262374_c0	Alpha-glucan phosphorylase, H isozyme	4.39	
comp265179_c0	Alpha-glucan water dikinase, chloroplastic	1.18	
comp259187_c0	Alpha-glucosidase 2	1.76	
comp108053_c0	Beta-glucosidase	4.02	
comp247530_c1	Glucan endo-1,3-beta-glucosidase 14	−1.16	
comp253693_c0	Glucose-6-phosphate 1-epimerase	1.08	
comp267337_c1	UDP-glucose:glycoprotein glucosyltransferase	1.13	
comp260021_c0	UDP-glycosyltransferase 74F2	2.81	
comp262276_c0	Xylulose kinase	1.80	
comp267262_c0	Beta-1,3-galactosyltransferase 15	−1.18	
comp176358_c0	Beta-galactosidase 15	1.39	
comp246181_c0	Vacuolar iron transporter homolog 5	3.32	Metal transporters
comp260577_c0	Aluminum-activated malate transporter 10	2.53	
comp268168_c1	ABC transporter B family member 1	1.86	
comp268748_c0	ABC transporter B family member 19	1.82	
comp262651_c1	ABC transporter B family member 19	3.75	
comp268004_c0	ABC transporter B family member 21	1.89	
comp269016_c0	ABC transporter C family member 9	2.15	
comp264448_c0	ABC transporter G family member 14	2.40	
comp258751_c0	Metal-nicotianamine transporter YSL12	2.15	
comp240449_c2	Nitrate transporter 1.5	2.51	Nitrate metabolism
comp228933_c0	Nitrate transporter 1.5	2.77	
comp268822_c0	Glutamate synthase 1 (NADH)	−1.21	
comp260213_c0	Glutamate dehydrogenase	3.08	
comp199170_c0	Metallothionein-like protein 1	1.51	Metal chelator
comp267212_c2	Nicotianamine synthase 1	7.27	
**(2) Zn-induced DEGs were not induced by Cd and Cd+Zn**			
**Unigene number**	**Annotation**	**Fold change^b^**	**Metabolism type**
comp232356_c0	Basic endochitinase A	−1.13	Carbohydrate metabolism
comp258906_c0	Pyruvate decarboxylase isozyme 2	1.13	
comp258084_c0	UDP-glycosyltransferase 73B3	1.41	
comp249756_c0	UDP-glycosyltransferase 73C4	−1.71	
comp242113_c0	Fructose-1,6-bisphosphatase	1.17	
comp262204_c0	Galactinol-sucrose galactosyltransferase	1.20	
comp262534_c1	Glucan endo-1,3-beta-glucosidase GII	−1.16	
comp231595_c0	Glucan endo-1,3-beta-glucosidase-like protein 2	2.31	
comp261591_c0	D-3-phosphoglycerate dehydrogenase	1.22	
comp254493_c0	Beta-fructofuranosidase, insoluble isoenzyme 7	1.18	
comp255444_c0	Mannose-6-phosphate isomerase	1.54	
comp257867_c0	5’-adenylylsulfate reductase 1	1.97	GSH metabolism
comp93164_c0	Glutathione S-transferase BZ2	2.52	
comp237801_c0	Glutathione S-transferase GSTU6	1.81	
comp248505_c0	Glutathionyl-hydroquinone reductase YqjG	1.01	
comp226841_c0	Ubiquinol oxidase 1a	2.38	Oxidation-reduction process
comp263260_c0	Ubiquinol oxidase 1a	1.62	
comp231302_c0	NADPH:quinone oxidoreductase 1	2.10	
**(3) Cd and Zn- induced DEGs were not induced by Cd+Zn**			
**Unigene number**	**Annotation**	**Fold change**	**Metabolism type**
comp262967_c0	Fructose 6-phosphate 1-phosphotransferase	−−	Carbohydrate metabolism
comp263404_c0	UDP-glycosyltransferase 85A2	−−	
comp239075_c0	Glutathione S-transferase GSTU6	−−	GSH metabolism
**(4) Cd and Cd+Zn- induced DEGs were not induced by Zn**			
**Unigene number**	**Annotation**	**Fold change^c^**	**Metabolism type**
comp268022_c1	Peroxidase 15	−1.56	Oxidation-reduction process
comp257839_c0	Catalase isozyme 2	1.99	
comp255896_c0	Polygalacturonase	2.53	Carbohydrate metabolism
comp258197_c0	Beta-glucosidase 4	1.84	
comp265016_c0	Nitrate transporter 1.5	1.17	Nitrate transporter
**(5) Zn and Cd+Zn- induced DEGs were not induced by Cd**			
**Unigene number**	**Annotation**	**Fold change^d^**	**Metabolism type**
comp216883_c1	NADH-ubiquinone oxidoreductase chain 2	2.38	Oxidation-reduction process
comp254393_c0	NADH-ubiquinone oxidoreductase chain 5	2.09	
comp258918_c0	NADH-ubiquinone oxidoreductase chain 5	2.23	
comp230435_c0	Mannose/glucose-specific lectin	−4.78	Carbohydrate metabolism
comp267969_c0	1-deoxy-D-xylulose-5-phosphate synthase 2	−2.50	
comp89835_c0	Glutathione S-transferase GSTF2	4.53	GSH metabolism
comp244563_c0	nitric oxide reductase	4.69	Nitrate metabolism
**(6) Cd+Zn-induced DEGs were not induced by Cd and Zn**			
**Unigene number**	**Annotation**	**Fold change^e^**	**Metabolism type**
comp259199_c0	Alpha-mannosidase 2	4.34	Carbohydrate metabolism
comp198796_c0	Beta-galactosidase 6	8.11	
comp257689_c0	Chitin elicitor receptor kinase 1	5.59	
comp250384_c0	Endochitinase A	3.12	
comp258505_c0	Beta-glucosidase 42	4.92	
comp125554_c0	Glucan endo-1,3-beta-glucosidase GV	−1.30	
comp263512_c0	Neutral alpha-glucosidase AB	−5.15	
comp245857_c0	Beta-amylase	4.16	
comp246661_c2	Callose synthase 12	5.01	
comp253875_c0	Callose synthase 3	5.52	
comp254996_c0	Callose synthase 9	5.55	
comp266662_c0	High affinity nitrate transporter 2.4	−1.15	Nitrate transporter
comp266662_c1	High affinity nitrate transporter 2.6	−1.20	
comp251323_c0	Cadmium-transporting ATPase	7.10	Metal transporter
comp254589_c0	Multidrug and toxin extrusion protein 1	6.26	
comp248484_c0	Plant cadmium resistance 4	6.86	
comp248297_c0	Nicotianamine synthase 9	−3.30	Metal chelator

a log2 Cd/CK;

b log2 Zn/CK;

c log2 Cd/CK;

d log2 Zn/CK;

e *log2 Cd+Zn/CK*.

### Validation of the expression of 12 selected DEGs

**Figure 4:**
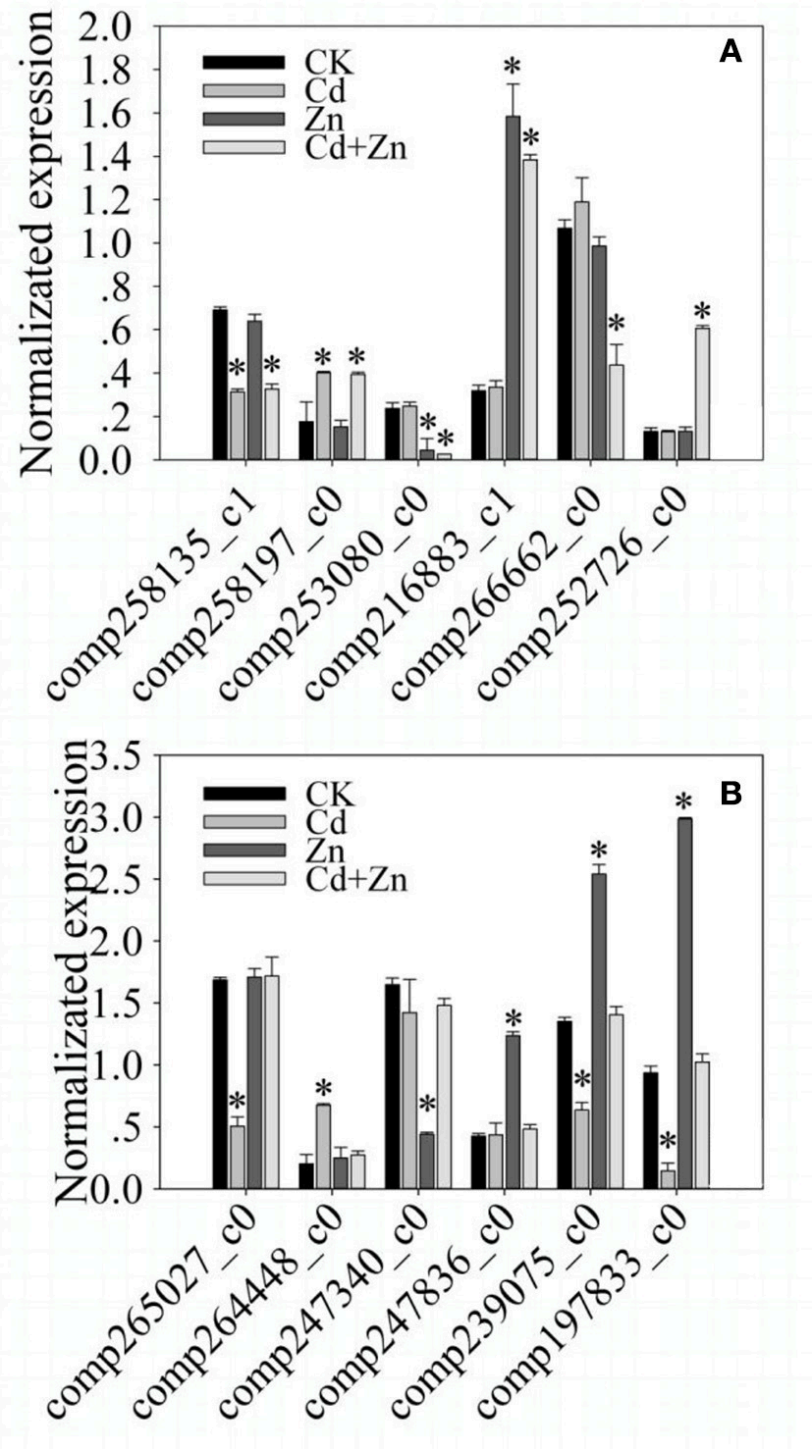
**qRT-PCR validation of the expression of randomly selected 12 DEGs that resulted from RNA-Seq. (A)** compared with CK, comp258135_c1 was down induced by Cd and Cd+Zn; comp258197_c0 was up induced by Cd and Cd+Zn; comp253080_c0 was down induced by Zn and Cd+Zn; comp216883_c1 was up induced by Zn and Cd+Zn; comp26662_c0 was specifically down induced by Cd+Zn; comp252726_c0 was specifically up induced by Cd+Zn. **(B)** comp265027_c0 was down induced by Cd; comp264448_c0 was up induced by Cd; comp247340_c0 was down induced by Zn; comp247836_c0 was up induced by Zn; comp239075_c0 and comp197833_c0 were down induced by Cd, but was up induced by Zn. Bars represented standard errors of three biological replicates. Asterisks represented significant differences between treatments and CK.

To validate different expression levels that resulted from RNA-Seq, the expression of 12 DEGs were normalized (STable 1). As shown in Figure 4, compared with CK, two DEGs were significantly (*P* < 0.01) regulated by Cd and Cd+Zn, but were not regulated by Zn (Figure 4A). Two DEGs were significantly (*P* < 0.01) regulated by Zn and Cd+Zn, but were not regulated by Cd (Figure 4A). Two DEGs were significantly (*P* < 0.01) regulated by Cd+Zn, but were not regulated by Cd and Zn (Figure 4A). Two DEGs were significantly (*P* < 0.01) regulated by Cd, but were not regulated by Zn and Cd+Zn (Figure 4B). Two DEGs were significantly (*P* < 0.01) regulated by Zn, but were not regulated by Cd and Cd+Zn (Figure 4B). Two DEGs were significantly (*P* < 0.01) regulated by Cd and Zn, but were not regulated by Cd+Zn (Figure 4B). These results were similar with the differential expression resulted from RNA-Seq, suggesting that DEGs resulted from RNA-Seq were credibly used to analyze the molecular responses to Cd, Zn, and Cd+Zn.

## Discussion

Cd inhibits the growth of bread wheat (Sun et al., 2005) and durum wheat (Hart et al., 2005; Koleva-Valkova et al., 2012). However, it also stimulates or does not inhibit the plant growth of other types of bread wheat (Stolt et al., 2003; Zhao et al., 2005; Lin et al., 2007). In the present study, although DPW accumulated 992.29 ± 29.83 mg/Kg (dry weight, DW) Cd in the roots and 40.82 ± 13.70 mg/Kg (DW) Cd in the shoots, the lengths of root and shoot were not affected (Figures 1A,B), which validated that DPW seedlings had stronger Cd tolerance than other types of durum and bread wheat (Hart et al., 2002, 2005). Meanwhile, the growth was inhibited by Zn and Zn+Cd stresses when the leaf and root accumulated high Zn concentrations (Figure 1), which indicated that excess Zn could cause the toxicity in wheat seedling (Zhao et al., 2005).

Since Cd and Zn mutually inhibited their uptake in the roots (Figure 2), the Cd/Zn interactions in the DPW roots were antagonistic, which was same as the antagonists in bread and durum wheat (Hart et al., 2002, 2005; Sun et al., 2005). However, Zn promoted the Cd transport from the roots to shoots (Figure 2B), Cd did not affect Zn transport (Figure 2D), which indicated that the Cd/Zn interactions in the DPW leaves were synergistic. This result was different from that Zn inhibited the Cd transport from the roots to shoots on 2 days after treatments with the same metal treated concentrations (Wang et al., 2016b).

Our previous study revealed that various proteins participated in the Cd/Zn interactions on 2 days after treatment (Wang et al., 2016b). In the present study, the transcriptomic changes in the roots also indicated that some genes were involved in the Cd/Zn interactions (Figure 3, STables 2–8). On 5 days after treatments, the expression of 1,269, 1,254, and 820 unigenes was individually changed by Cd, Cd+Zn, and Zn. However, only 381 unigenes were co-regulated by these treatments (Figure 3). In addition to the specific unigenes individually induced by Cd, Zn, and Cd+Zn, the remaining DEGs classified into different subgroups were considered to participate in the Cd/Zn interactions. These results indicated that Cd, Zn, and Cd+Zn induced differential molecular responses, which ultimately resulted in the differential molecular responses for Zn and Cd stresses (Lin and Aarts, 2012). In the following discussion, some DEGs involved in several important processes were described.

Heavy metal transporters play important roles in the metal uptake, transport and distribution. In yeast, the expression of cadmium-transporting ATPase which is a cadmium-specific efflux pump enhanced Cd resistance by extruding intercellular Cd (Adle et al., 2007; Adle and Lee, 2008). In the DPW roots, the expression of *cadmium-transporting ATPase* and *plant cadmium resistance* 4 was specifically up-regulated by Cd+Zn (Table 1), suggesting that Cd might be extruded from roots, finally resulted in that the Cd concentration in the roots under Cd+Zn stress was significantly lower than that under Cd stress (Figure 2A). Therefore, Zn can enhance the Cd resistance by reducing the Cd accumulation (Rizwan et al., 2016).

Except of metal efflux pumps, other heavy metal transporters also play important roles in heavy metal detoxification. In *Arabidopsis*, AtABC25 (Kim D. Y. et al., 2006) and AtABCC1-3 (Bovet et al., 2003, 2005; Park et al., 2012; Brunetti et al., 2015) transported Cd into the vacuoles to increase Cd tolerance. Overexpression of a metal chelator, *metallothionein* (*MT*), increased Cd content in the roots and enhanced Cd tolerance (Sekhar et al., 2011). In this study, *6 ABC transporters* (B, C, and D family members, Table 1) and a *MT* were up-induced by Cd, suggesting that most of Cd accumulated by root might be sequestrated into the vacuoles so that DPW seedling exhibited strong Cd tolerance under Cd stress (Figure 1). When these regulations were not observed under Cd+Zn stress (Table 1), the Cd accumulations in the roots should be reduced (Bovet et al., 2003, 2005; Sekhar et al., 2011; Park et al., 2012; Brunetti et al., 2015), finally resulted in that the Cd concentration in the roots under Cd stress was higher than that under Cd+Zn stress (Figure 2A). Meanwhile, most of Cd accumulated by the roots was not sequestrated into the vacuoles, but was uploaded into the xylems and then transported into the shoots (Figure 2B, Kim et al., 2007). Thus, these *ABC transporters* and *MT* participated in the Cd/Zn interactions for the Cd transport and accumulation.

In *planta*, the high accumulation of Fe in the shoots under Cd stress could alleviate Cd toxicity (Wu et al., 2012). Overexpression of *nicotianamine synthase* (*NAS*), *vacuolar iron transporters* (*VIT*), and *metal-nicotianamine transporter YSL* (*YSL*) increased the Fe content in the roots and shoots (Kim S. A. et al., 2006; Ishimaru et al., 2010; Wu et al., 2012). In the present study, Cd stress through up regulating *NAS1, VIT*, and *YSL12* (Table 1) to increase the Fe content in the DPW roots and shoots (data not shown), which could alleviate Cd toxicity (Wu et al., 2012), and then enhanced the Cd tolerance in DPW seedlings (Figure 1). However, these regulations were not observed under Zn and Cd+Zn stresses (Table 1), suggesting that these up regulations were activated when the roots accumulating a certain amount of Cd.

Cd inhibits the nitrate assimilation and transport in *planta* (Sanita di Toppi and Gabbrielli, 1999; Li et al., 2010). Cd regulates several nitrate-related genes, such as *glutamate dehydrogenase* and *nitrate transporter* (NRT) (Chaffei et al., 2004; Li et al., 2010), and also affects leaf nitrogen remobilization and root nitrogen storage (Chaffei et al., 2004). Down-regulation of *AtNRT2.8* reduced the Cd accumulation in the roots and increased it in the shoots. Thus, *AtNRT2.8*-regulated nitrate distribution controls the Cd uptake and transport (Li et al., 2010). In the present study, Cd alone up regulated *glutamate dehydrogenase* and two *NRTs* (Table 1), suggesting that Cd might regulate the nitrate assimilation and transport in the DPW roots (Chaffei et al., 2004). Meanwhile, two high affinity nitrate transporters (*HANTs*) were specifically down-regulated by Cd+Zn (Table 1). *HANTs* participate in the nitrate uptake (Cerezo et al., 2001; Li et al., 2007). The inhibition of nitrate uptake reduces the Cd uptake and other essential metals (Mao et al., 2014). Thus, down regulation of *HANTs* might mutually inhibit the Cd/Zn uptake under Cd+Zn stress (Table 1). Due to different nitrate-related genes were induced, Cd and Cd+Zn might regulate different nitrate metabolism.

Although these heavy metal and nitrate transporters were not observed at proteomic level on two days after treatments (Wang et al., 2016b), unigenes participated in glutathione (GSH) metabolism, antioxidant enzymes and cell wall composition were induced at both transcriptomic (Table 1) and proteomic level (Wang et al., 2016b). GSH is a substrate for phytochelatin synthesis and crucial for detoxification of heavy meals (Freeman et al., 2004; Yadav, 2009). Formation of GSH-Cd or Zn complexes and then sequestration into the vacuoles is another mechanism of detoxification (Seth et al., 2012; Jozefczak et al., 2015). Genes involved in GSH metabolism, such as *glutathione S-transferase* (*GST*), *hydroxyacylglutathione hydrolase* (*HGH*), and *glutaredoxin* (*Grx*), were differentially regulated by heavy metals (Di Baccio et al., 2011; Lin et al., 2013; Jozefczak et al., 2015). As detoxifying enzymes present in all aerobic organisms, GSTs catalyze the nucleophilic attack of the sulfur atom of the tripeptide GSH on the electrophilic group of the substrate (Adamis et al., 2004), and also transport compound of GSH-cytotoxic substrates into the vacuoles for detoxification (Kumar et al., 2013). In this study, several unigenes of GSH metabolism, such as *Grx, HGH, lactoyglutathione lyase, S-formylglutathione hydrolase, disulfide isomerase-like 1-4*, and 5 *GSTs*, were induced and grouped into different interactions of Cd/Zn (Table 1), suggesting that GSH metabolism played different roles in the Cd/Zn interactions (Wang et al., 2016b).

Plants have established effective antioxidative systems to protect cells against damage from metal-induced oxidative threats (Di Baccio et al., 2011). Previous studies revealed that many Cd or Zn- induced genes participated in the defense against oxidative stress (Di Baccio et al., 2011; Lin et al., 2013). In this study, 13 *peroxidases* (*POD*) and 3 *aldehyde dehydrogenases* that removed the toxic aldehydes from lipid peroxidation were regulated by Cd, but were not regulated by Zn and Cd+Zn stresses (Table 1). Three genes (two *ubiquinol oxidase 1as* and *NADPH: quinone oxidoreductase 1*) were up-regulated by Zn but were not induced by Cd and Cd+Zn stresses (Table 1). *Catalase* (*CAT*) *isozyme 2* was specifically up-regulated by Cd (Table 1). These results confirmed that antioxidant enzymes play different and important roles in adaptive response to Cd, Zn, or Cd+Zn stresses (Qiu et al., 2008; Zeng et al., 2011).

The plant cell wall is mainly composed of cellulose and polysaccharides (Cosgrove, 2005). It can be modified by Cd (Li et al., 2015; Shi et al., 2015). Therefore, modification of cell wall composition is associated with the Cd exclusion in the roots (Zhu et al., 2012). Additionally, exogenous glucose also alleviates the Cd toxicity by fixing Cd in the cell wall and sequestering it into the vacuoles (Shi et al., 2015). In this study, many carbohydrate (including glucose, cellulose, callose and mannose) metabolism-related genes were differentially regulated by Cd, Zn, or Cd+Zn (Table 1), which suggested that cellulose, glucose and polysaccharides play different roles in the Cd and Zn fixation, exclusion and sequestration in the roots (Li et al., 2015).

## Author contributions

YW, XW, CW, and YZ conceived and designed research, and wrote the manuscript. YW, XW, CW, XX, FP, and RW conducted experiments. YW, XW, JZ, HK, XF, LS, and HZ analyzed data. All authors read and approved the manuscript.

### Conflict of interest statement

The authors declare that the research was conducted in the absence of any commercial or financial relationships that could be construed as a potential conflict of interest.
